# Effect of gas injection on cavitation-assisted plasma treatment efficiency of wastewater

**DOI:** 10.1016/j.ultsonch.2022.105941

**Published:** 2022-02-04

**Authors:** Yifan Xu, Takuya Yamamoto, Daiki Hariu, Sergey Komarov

**Affiliations:** a Department of Frontier Sciences for Advanced Environment, Tohoku University, 980-8579 Sendai, Japan; b Department of Metallurgy, Tohoku University, 980-8579 Sendai, Japan

**Keywords:** Underwater plasma, Ultrasound irradiation, Acoustic cavitation, High-voltage pulse, Gas injection, Rhodamine B degradation

## Abstract

Underwater plasma has been long recognized as a “green” alternative to conventional chemicals in the wastewater treatment processes. However, practical application of underwater plasma is still challenging due to insufficient treatment performance. Recently, we proposed a novel process named ACAP utilizing acoustic cavitation in order to stabilize the plasma generation and to enlarge the plasma processing region.

This work continues our investigation regarding the ACAP treatment process focusing on effects of gas injection. Experiments were performed using an ultrasonic installation equipped with a specially designed sonotrode (Diam. 48 mm) operated at a frequency of 20 kHz and acoustic power of 120 W. The results revealed that the degradation efficiency of Rhodamine B, which was used as a model wastewater pollutant, remains almost unchangeable in the case of air injection, but it is doubled when argon is injected into the ACAP reactor. It was found that the argon injection enhances the degradation efficiency significantly even without ultrasound irradiation. Results of additional measurements suggest that the effect of argon is attributed to its ability to yield high temperature during cavitation, comparatively good solubility in water and a better ability to reduce the breakdown voltage in water compared to the air case.

## Highlights

• Investigation on gas injection during ACAP process is conducted.
• Argon provides a better efficiency of Rhodamine B degradation compared to air.
• Hydrogen and oxygen-containing radicals are generated in larger amount during argon injection.
• Argon dissolves in water that lowers breakdown voltage and improves cavitation treatment.

## 1 Introduction

Recently, underwater plasma has received much attention as a “green” alternative to conventional chemicals in wastewater treatment technology. Starting from the pioneering work of Clements et.al [1] in 1987, a great number of studies has been made to investigate the efficiency of underwater plasma treatment including organic pollutants, pharmaceutical residues and various pathogens. Typically, underwater plasma is produced due to the electrical breakdown of water by applying high-voltage pulses in the interelectrode space. The major mechanisms by which underwater plasma exerts its effects on water pollutants have been also a subject of extensive studies in the past [2], [3], [4]. The mechanisms can be subdivided into two categories. This first one is a thermal action on chemicals and pathogens, causing their direct thermal decomposition near or inside high-temperature plasma channels. This mechanism is especially important when pollutants are hydrophobic and/or volatile. Since typically the concentration of pollutants in wastewater is small and most of them are hydrophilic and non-volatile, their degradation occurs according to the second mechanism involving chemical reactions with highly active radicals like H⋅, OH⋅ and O⋅. These radicals can be generated through a number of mechanisms, with the main contributors believed to be thermal decomposition of water in the high-temperature plasma channels and collision with high energy electrons in the space between electrodes. In either case, the lifetime of these radicals is extremely short, suggesting that they cannot be transported far from the generation area.

Thus, as all the above-mentioned phenomena take place in the area near or between electrodes, the mass transfer of pollutants to this area plays the extremely important role in their treatment efficiency. This is the main reason why so many techniques of plasma treatment have been proposed and evaluated in the past. Although in most cases, no consideration was given to the mass transfer phenomena in the earlier studies, a great variety of designs proposed for the plasma treatment equipment and strong dependence of the treatment efficiency on the equipment design features suggest that the mass transfer and its enhancement play the dominant role in improving the performance of underwater plasma processes. A lot of relevant information on the design and efficiency of various treatment techniques can be found in recent review papers, for example [2], [5], [6], [7]. Despite the variety of technical solutions proposed, there is one common feature that they share, and it consists in the fact that an introduction of gas bubbles into the space between electrodes was found to be very effective for reducing the breakdown voltage and stabilizing the pulse discharge in water or its solution.

Based on the above findings, in our previous research, we proposed a novel technique of wastewater processing, acoustic cavitation assisted plasma (ACAP), which combines the high-voltage pulse discharge and acoustic cavitation [8], [9]. The main idea was to exploit two phenomena occurring when ultrasound vibrations are introduced in liquids, namely acoustic cavitation and acoustic streaming. The first phenomenon produces a tremendous amount of tiny bubbles in the cavitation zone. It is expected that these bubbles can serve as sites at which electrical breakdown can occur more readily, reducing, thus, the breakdown voltage. Acoustic streaming is a steady flow created near the ultrasonic sonotrode as a result of attenuation of acoustic energy in the cavitation zone. Depending on the vibration amplitude, the velocity of acoustic streaming can be as high as 0.4 m/s [10], [11]. Obviously, such a fast flow can improve mixing and mass transfer of pollutants in the plasma treatment reactor. More details on these phenomena can be found in our earlier papers [8], [10], [11]. In the following investigations, it was found that the acoustic streaming always has a beneficial effect on the efficiency of plasma treatment in both batches [9] and circulatory [8] reactors. As for acoustic cavitation, its effect on the plasma treatment efficiency is more complicated. High-speed camera observations showed that pulse discharge occurs more often during the rarefaction half-cycle of ultrasound wave than during the compression half-cycle [8]. This is because cavitation bubbles are nucleated and grow rapidly when the surrounding pressure becomes low. On the other hand, as found in another our study [9], pulse discharge and plasma channel formation start when comparatively large bubbles with the size of about 1 mm approach the high voltage electrode. Although the bubbles in cavitation zone can grow and coalesce with each other, the vast majority of cavitation bubbles in water have much smaller sizes. Furthermore, a prolonged irradiation of ultrasound in liquid causes degassing of water that can result in a gradual reduction of the concentration of cavitation bubbles in the interelectrode space. Therefore, the above findings suggest that an additional introduction of gas in the reactor could be beneficial to enhance the cavitation activity of bubbles and, thus, to improve the efficiency of plasma processing.

Thus, the goal of the present paper is to examine the effect of gas injection on the degradation efficiency in the ACAP process. In the experiments, air or argon was injected into the ACAP reactor through L-shaped nozzles or a porous plug, and Rhodamine B was used as a model contaminant. The same experiments were performed under the plasma-alone and ultrasound-alone treatment conditions. The experimental results obtained are discussed from the point of view of gas characteristics, behavior of bubbles and pulse discharge frequency.

## 2 Experimental

### 2.1 Experimental setup

**Fig. 1:**
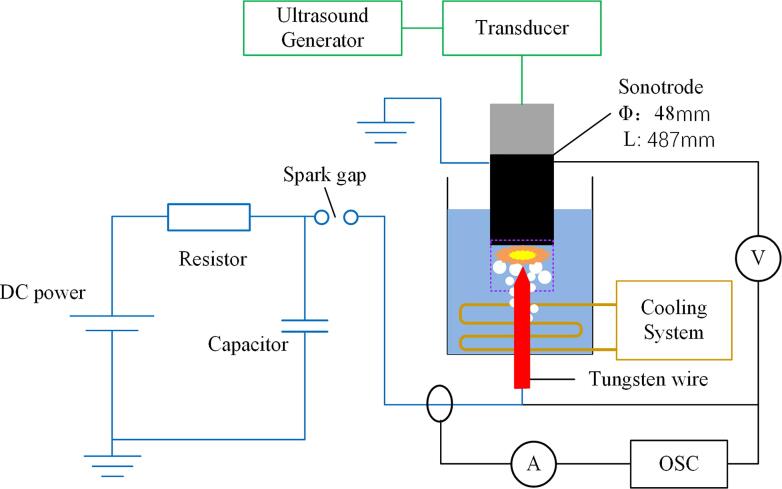
Schematic drawing of experimental setup.

The experimental setup used was the same as that in our previous work [9]. Its schematic drawing is shown in Fig. 1. For a better understanding of the following explanations, a brief description of the setup is given below.

A DC power source (HAR-40P7.5-LN, Matsusada, Japan) with maximum power of 300 W and voltage of + 40 kV was applied to charge a 1000 pF ceramic capacitor and generate pulses first through a spark gap unit and then through a needle-plate electrode system installed inside an acrylic cylindrical vessel filled with two liters of a Rhodamine-B aqueous solution. The vessel had an inner diameter of 13 cm and a height of 30 cm. The high-voltage needle electrode, made of 1 mm tungsten wire, was fixed vertically under the plane tip of an ultrasound sonotrode, which served as a grounded electrode. The distance between electrodes was 4 mm. All experiments were performed under a voltage of 26 kV. A water-cooled coil was submerged in the vessel to maintain the water temperature at 20 ± 2 °C.

**Fig. 2:**
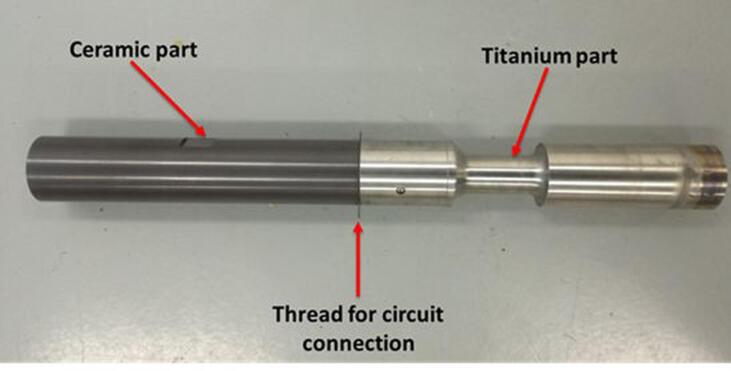
Appearance of ultrasonic sonotrode.

An ultrasonic generator (WS-1200–28, Honda Electronics, Japan) was applied to irradiate ultrasound vibrations into the water tank at a frequency of 20 kHz. Ultrasound vibrations were transmitted into water through a cylindrical dumbbell sonotrode of 48 mm in diameter and 487 mm in length connected to a piezoelectric ceramic transducer. The ultrasonic sonotrode consisted of two cylindrical parts, one made of Si_3_N_4_ to prevent high voltage damage of the ultrasonic generator and the other made of titanium. Both parts are shown in Fig. 2. The titanium part also served as the ground electrode. The dimensions of both parts were adjusted to resonant conditions. The amplitude of sonotrode tip was 36 μm (p-p), that significantly exceeds the threshold of developed cavitation onset in water which is 4–5 μm (p-p) [12]. Acoustic power was measured by a calorimetric method. Under the present experimental condition, the acoustic power was 120 W.

In addition, some experiments were conducted with the use of a highspeed video camera (FASTCAM1024PCI, Photron, Japan) to take images at a shutter speed of 1/3000 and a frame speed of 3000 per second. Plasma emission spectra generated underwater conditions were examined by emission spectroscopy using a multiband plasma process monitor system (C10346-01, Hamamatsu Photonics K.K., Japan). The measurement wavelength range was from 200 nm to 950 nm with a resolution of 2 nm.

### 2.2 Experimental procedure

Rhodamine B (Wako Pure Chemical Co., Japan) was used as a model contaminant. Influences of conductivity, pH and initial concentration of Rhodamine B on its decomposition efficiency have been studied in our previous study [9]. Therefore, based on those results, in the present study the solution pH and conductivity were adjusted to 5 and 100 µS/cm, respectively using the appropriate NaCl or H_2_SO_4_ solutions. The initial concentration of Rhodamine B (hereinafter RhB) was set to 5 mg/L.

The experiments were conducted with and without gas injection. Each experiment continued for 12 min, during which solution samples were taken every 3 min to measure the RhB concentration using a spectrophotometer (UV–VIS) and thus to determine the RhB decomposition efficiency, η which was calculated as follows:

(1) $$\eta =100\times \left\{{\left[\mathrm{RhB}\right]}_{0}-\left[\mathrm{RhB}\right]\right\}/{\left[\mathrm{RhB}\right]}_{0}$$

where [RhB] is the concentration of RhB after the treatment, [RhB]_0_ is the initial concentration. Besides, concentration of H_2_O_2_ was measured using a hydrogen peroxide meter (H_2_O_2_-V3, Kasahara Chemical Instruments Corp., Japan).

**Fig. 3:**
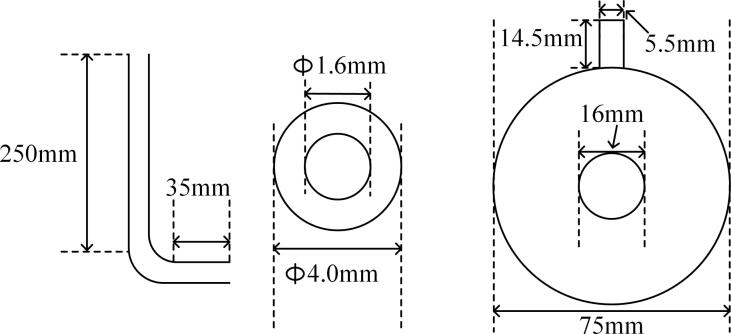
Bubble injection unit: (a) glass nozzles (b) porous plug.

Air or argon were injected into the reaction vessel through L-shaped glass nozzles or a porous plug, as described below. The gas flow rate was set to 2, 4, 6 or 8 L/min. The main dimensions of nozzle and porous plug are shown in Fig. 3.

**Fig. 4:**
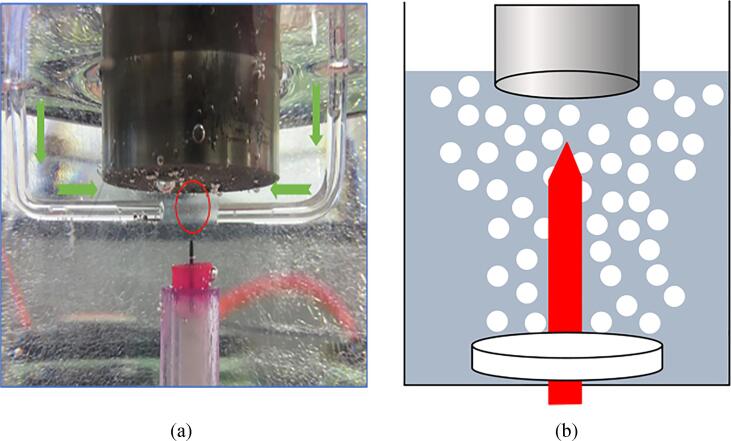
Bubble injection through (a) glass nozzles (b) porous plug.

The L-shaped glass nozzles were fixed close to the sonotrode tip to inject gas into the zone of plasma generation, as shown in Fig. 4(a). The porous plug was placed on the vessel bottom in order to produce much smaller bubbles compared to the L-shaped nozzle case. This is schematically shown in Fig. 4(b).

Besides, some additional experiments were conducted to elucidate the effects of key parameters on the RhB degradation efficiency in more details. Procedure and conditions of these experiments will be described in the following sections.

The emission spectra of underwater plasma were analyzed by emission spectroscopy. In order to prevent the influence of daylight, the experiment was carried out in complete darkness. The exposure time was set as 200 ns to take a spectrum set under typical experimental conditions. Typically, such a spectrum set comprised 10 ∼ 15 spectrum taken at different moments of plasma discharge development.

## 3 Results

### 3.1 Effect of gas type and injection method on decomposition efficiency

**Fig. 5:**
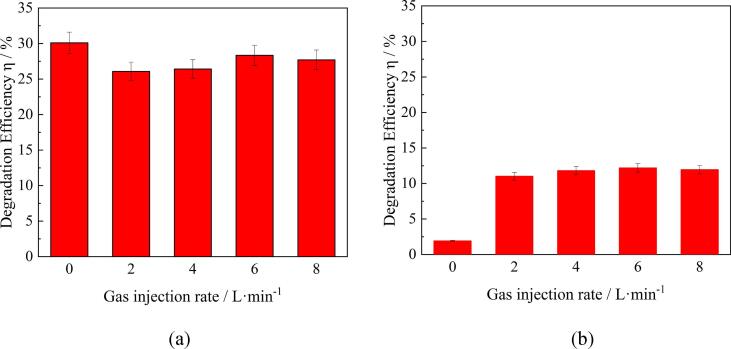
Effect of air flow rate on RhB decomposition efficiency when glass nozzles were used: (a) ACAP treatment, (b) plasma alone.

Fig. 5 shows the effect of gas flow rate on the RhB decomposition efficiency under different experimental conditions using the L-shaped nozzles for air injection. It is to be noted that a comparison of decomposition efficiency in this and the following bar charts was performed after 12-min treatment experiments. When plasma is used in combination with ultrasound irradiation, the decomposition efficiency is slightly decreased with increase of air flow rate, as can be seen from Fig. 5(a). These data suggest that there is very little or no effect of air injection on the degradation efficiency. On the other hand, the air injection resulted in a significant enhancement of degradation efficiency in the experiments using plasma treatment alone. As can be seen from Fig. 5(b), the degradation efficiency is only about 2% without gas injection and ultrasound irradiation. Then, the decomposition efficiency is drastically increased with the gas injection and kept almost constant, being approximately 12%. Therefore, a comparison of Fig. 5(a) and (b) reveal that the ultrasonic irradiation causes a great improvement in the decomposition efficiency, however, injection of air through the glass nozzles is ineffective in this case.

**Fig. 6:**
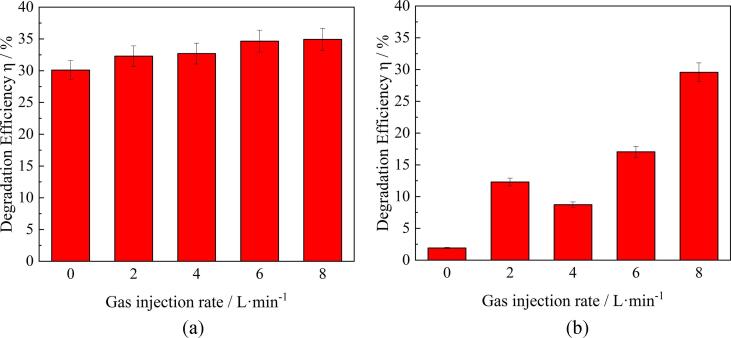
Effect of air flow rate on RhB decomposition efficiency when the porous plug was used: (a) ACAP treatment, (b) plasma alone.

When gas was injected through the nozzles, the diameter of bubbles was found to be relatively large. Such bubbles can hinder the propagation of ultrasonic waves preventing the generation of cavitation bubbles. To further investigate the effect of gas bubbles, a series of experiments was conducted to generate finer bubbles through the porous plug. Fig. 6 shows the decomposition efficiency when air gas was injected through the porous plug under conditions of ACAP treatment (a) and plasma alone (b). The results reveal that, in this case, the effect of air injection on the RhB decomposition efficiency is small in the ACAP treatment but becomes larger when the plasma alone is used for the treatment. This result is very similar to that obtained with the glass nozzles. However, in general, the porous plug yielded a greater effect compared to the L-shaped glass tubes. For example, at the maximum gas flow rate of 8 L/min, the degradation rate under the ACAP treatment conditions was equal approximately to 33% when gas was injected through the porous plug, and 28% in the case of tube injection. When the plasma treatment was performed without ultrasound, this difference becomes yet larger. At the maximum gas flow rate, the degradation efficiency did not exceed 12% when gas was injected through the tubes, while it is increased up to 30% when the porous plug was used.

**Fig. 7:**
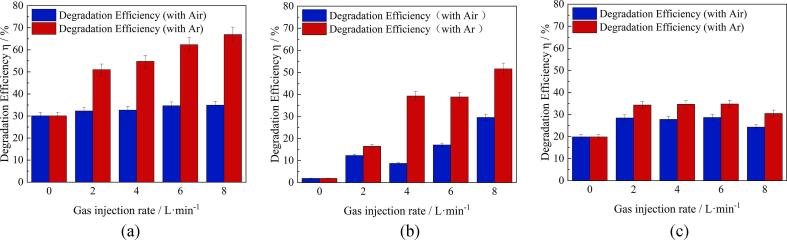
Effect of gas flow rate on RhB decomposition efficiency: (a) ACAP treatment, (b) plasma alone treatment, (c) ultrasonic alone treatment.

Fig. 7 shows the degradation efficiency under various conditions when argon was injected through the porous plug. Generally, the degradation efficiency of RhB with argon gas is higher than that with air, reaching 68% in the ACAP treatment (Fig. 7a) and 50.6% in the plasma alone case (Fig. 7b) at the gas flow rate of 8 L/min. As can be seen from Fig. 7(a), under the ACAP treatment, the degradation efficiency is gradually increased with Ar gas flow rate, while during the air injection, the degradation efficiency remains almost constant. Treatment with plasma alone was also much effective under the argon injection compared to the air injection case, as seen from Fig. 7(b). As for the ultrasonic alone treatment (Fig. 7c), it is clearly seen that the decomposition efficiency first increases with the argon injection, and then it is kept almost constant or slightly decreases as the gas flow rate increases. Besides, similar to the ACAP and plasma alone treatments, argon has a better effect on the decomposition efficiency than air.

### 3.2 Effect of gas type on breakdown voltage and plasma discharge frequency

Thus, the above results reveal that the decomposition efficiency of RhB becomes greater in the presence of argon as compared to the air injection case. To investigate possible mechanisms in more details, we conducted the following additional measurements: 1 – breakdown voltage, 2 – plasma discharge frequency.

To measure the breakdown voltage, air or argon was injected into a water bath through the porous plug for 3 min, and then the voltage between the electrode was gradually increased until the moment of the first discharge. These measurements were conducted without ultrasound application. The voltage at this moment was considered as the breakdown voltage. In order to ensure that 3-min injection was sufficient to saturate the water bath with gas, concentration of dissolved oxygen was measured using an oxygen meter (HORIBA, OM-51). The initial concentration of oxygen in water was 5.73 mg/L. After injection of air, the oxygen concentration remained unchangeable and equal to 5.66 mg/L. However, after Ar injection, the oxygen concentration reduced to 0.41 mg/L. Thus, these results indicate that, first water was saturated by oxygen initially and, therefore, the air injection does not affect the oxygen concentration.

Table 1 shows the measurement results of breakdown voltage. It is seen that the argon injection provides a slightly lower value of the breakdown voltage compared to the air injection. It is interesting to compare values of breakdown voltage for air and argon in gaseous state. According to the relevant literature [13], the electric strength of air and argon is 35.5 kV/cm and 7.2 kV/cm, respectively. This difference is much greater than that obtained when these gases were injected in water. This is because both air and argon exist in water, either in a dissolved state or as microbubbles, and their amount in either case is very small.

**Table 1:** Change in dielectric breakdown voltage due to dissolved gas.

Dissolved Gas	Ar	Air	No Gas
Breakdown voltage (kV)	23.4	24.8	27.2

Measurements of plasma pulse frequency were performed at the same conditions as those of RhB decomposition experiments, namely a voltage of 26 kV, solution pH of 5 and conductivity of 100 µS/cm under the following conditions of gas injection through the porous plug: (1) continuous injection of gas at 4 L / min during plasma discharge (2) injection of gas during 3 min at 8 L / min and plasma discharge after stopping the injection. The video was recorded for 3 s to evaluate the average frequency of pulse plasma discharge. The measurement results are summarized in Table 2.

**Table 2:** Frequency of plasma discharge under various conditions, Hz.

	Gas Type	With Ultrasound	Without Ultrasound
Continuous injection at 4 L/min	Ar	37.5	35.4
	Air	31.0	6.2
Prior injection at 8 L/min followed by plasma discharge	Ar	46.0	37.9
	Air	43.7	0
Without Gas	No Gas	26.5	0

It is seen that the discharge frequency during argon injection is larger than that during air injection regardless of injection conditions. The difference in the average frequency between these two gases becomes greater when the plasma is generated without ultrasound irradiation, especially in the case of injection interruption. Observations showed that bubbles are absent in the space between the electrodes after the gas injection interruption. However, fine invisible bubbles can still exist in the solution affecting the plasma discharge frequency. Thus, it is difficult to separate the effects of dissolved gases and bubbles on the breakdown voltage and discharge frequency. Nevertheless, additional consideration suggests that the dissolved gas and its properties could play the main role in the improvement of degradation efficiency. Some details are given in the discussion section.

## 4 Discussion

Based on the results presented in the previous sections, one can conclude that the effect of ultrasound irradiation, when compared to that of plasma-alone treatment, is varied with the gas type and its injection technique. Moreover, the gas type was found to affect the decomposition efficiency drastically. Below is an explanation of the effects obtained and possible mechanisms based on the above experimental results.

### 4.1 Effect of gas injection method

Under most conditions examined, injection of gas into the reactor improved the decomposition efficiency however the improvement degree was different in the ACAP, plasma-alone and ultrasound alone treatments, and depended on the gas type, flow rate and injector design. Generally, injection of air through the L-shaped glass nozzles was the least efficient. As can be seen from Fig. 5(a), the decomposition efficiency is even decreased in the ACAP process during the air injection, especially at smaller flow rates. At least, two reasons can be responsible for such a variation. First, the gas injection through the glass nozzles produces relatively large bubbles. Initially, the bubbles were expected to be fragmented in the cavitation zone and carried out by the acoustic streaming into the space between the electrodes. However, observations of the bubble behavior revealed that the bubbles escape from the cavitation zone before fragmentation, not approaching the high-voltage electrode. This was also confirmed by the results of numerical simulation, as presented below. On the other hand, in the plasma-alone treatment, the air injection is very effective in increasing the decomposition efficiency, as can be seen from Fig. 5(b). Injection of air underneath the sonotrode tip through the glass nozzles can result in dissolution of air in water in the immediate vicinity of the high voltage electrode. Furthermore, as there is no acoustic streaming, some air bubbles can enter the space between electrodes. Both these phenomena should cause the breakdown voltage to decrease as shown in Table 1.

When air was injected through the porous plug, a much greater decomposition effect was obtained. Obviously, compared to the glass nozzles, the porous plug allows one to produce much finer gas bubbles which rise slowly in the solution bath and, therefore, can exist in water much longer than large bubbles. Diameter and rising velocity of typical bubbles, measured by the video camera at a flow rate of 8 L/min, were around 2–3 mm and 0.2 m/s, respectively. Due to the low rising velocity, small bubbles can be easily entrained into a liquid flow in the reactor and enter the space between the electrodes. Besides, small size of bubbles is favorable for gas dissolution because of wider contact area between the bubbles and water. Thus, both the presence of small bubbles and dissolved gas can contribute to the improvement of RhB decomposition efficiency.

However, as results of numerical simulation revealed, even small bubbles cannot enter in the space between electrodes due to a strong radiation pressure acting as the bubbles approach the sonotrode tip. The results are presented below.

### 4.2 Effect of gas type and flow rate

As a result of the presence of gas, whether it be dissolved or dispersed as fine bubbles in the space between electrodes, the plasma pulse discharge occurred more often in the experiments with gas injection, as shown in Table 2. The greatest effect of gas injection was found when the solution was treated with the plasma alone. Under these conditions, the argon injection was much more efficient than the air injection. As seen from Table 2, injection of air was ineffective at all when air was injected prior to the pulse plasma discharge and little effect during injection simultaneously with the pulse discharge. On the other hand, the argon injection provided the pulse discharge at frequencies higher than 30 Hz under both conditions of gas injection. A number of reasons can be responsible for such an effect, as considered below.

As shown above, the breakdown voltages of water after injection of air and argon are 23.4 kV and 24.8 kV, respectively. Although the difference between these values in absolute terms is quite small, subtracting these values from 26 kV, which corresponds to the voltage applied in the RhB degradation experiments, yields 2.6 kV and 1.2 kV, respectively. This indicates that, in the experiments using argon, the value, by which the voltage between electrodes exceeds the breakdown value, is approximately twice greater than that in the experiments using air. As mentioned above, this is because gaseous argon itself has a much lower breakdown compared to the air case. One more possible reason is that solubility of Ar in water is higher than that of air. For example, at room temperature the solubility, expressed in molar fraction, is 2.748 × 10^-5^ for argon and 1.524 × 10^-5^ for air [14]. Obviously, under these conditions, the pulse discharge frequency and the RhB degradation efficiency in the case of argon injection should be higher as compared to the air injection case. From this point of view, it is clear why the degradation efficiency is improved as the argon flow rate becomes larger in the case of ACAP and plasma alone treatments.

In addition, there is one more reason why the degradation efficiency is low when air is injected into the RhB solution. It is well known that nitrogen and oxygen can chemically react at high temperature including cavitation hot spot [14] and underwater plasma [15], [16]. As a result, a number of nitrogen oxides are produced, and some of them can capture OH⋅ radical to form nitrite or nitrate ions at the gas–liquid interface followed by their dissolution in water [15]. Typical OH⋅-capturing reactions can be expressed as follows:

NO_2_ + OH⋅ → H^+^ + NO^-^_3_

NO + OH⋅ → H^+^ + NO^-^_2_

Other ion-forming reactions also can occur in the presence of air in the system. Their details can be found in the relevant literature [16]. As documented in our earlier paper [9], the pulse frequency of plasma discharge lowers with increasing the solution ionic conductivity. This is because ionic current occurs between the electrodes in the presence of dissolved ions causing the breakdown voltage to increase. Thus, both the OH radical capture and breakdown voltage increase can cause the reduction of the RhB degradation efficiency under the air injection conditions as compared to the argon injection case. Inefficiency of air injection especially stands out when the RhB solution is treated with the ACAP and ultrasound alone process, as seen from Fig. 7(a) and (c).

**Fig. 8:**
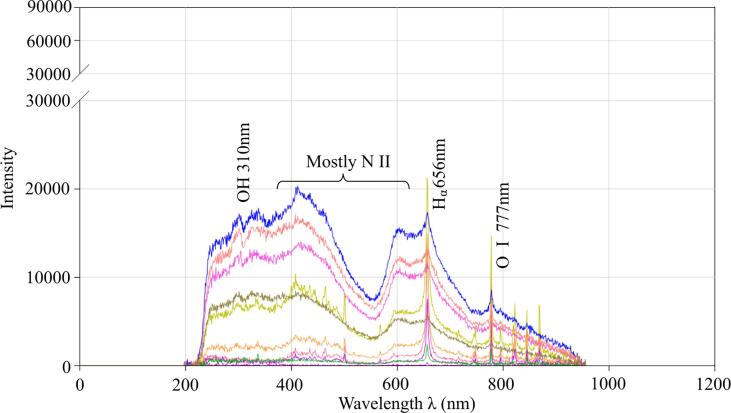
The spectrum under the condition of Air injection.

**Fig. 9:**
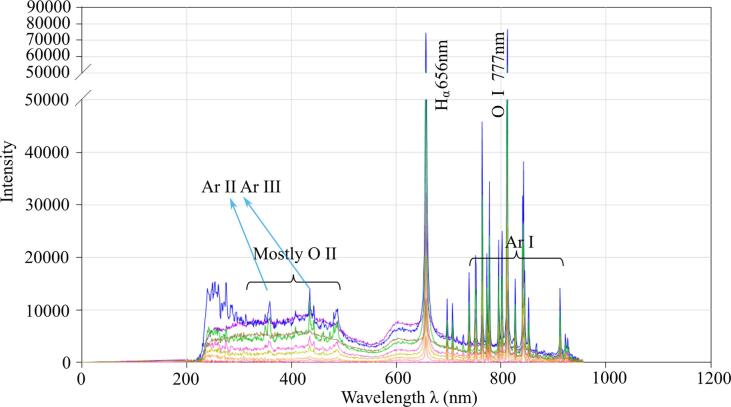
The spectrum under the condition of Ar injection.

The presence of nitrogen-containing chemicals can be confirmed by emission spectra taken during the plasma discharge with gas injection. Fig. 8, Fig. 9 present typical spectra obtained when air or argon was injected regardless of whether ultrasound was applied or not. Experimental details have been explained above. Both figures reveal that the spectra consist of two parts: continuous spectra where the intensity is smoothly varied between small peaks (Fig. 9) or without peaks (Fig. 8) in the wavelength range from 200 nm to 600 nm, and mixed spectra where low (Fig. 8) or tall (Fig. 9) peaks are superimposed on the continuous part when the wavelength exceeds 600 nm. It is clearly seen that, in the case of air injection, the emission intensity of continuous part of the spectra is significantly larger compared to the case of argon injection. Although the occurrence of such continuous spectra can be caused by a variety of species, the data of the relevant literature show that the contribution of oxygen or nitrogen-containing ions and radicals such as N II and O II [17], [18] can be significant. Locations of some of these peaks are shown by vertical lines in Fig. 8.

In the mixed parts of spectra, the opposite tendency is found. When argon gas is injected into water, several tall peaks are clearly observed in Fig. 9, while in the case of air injection, some peaks are although observable in Fig. 8, but of much smaller intensity. Identification of these peaks suggested that they are originated from such elements as hydrogen, oxygen and argon. The first one exists as Hα [19], whether air or argon injection is used, with the most pronounced peak at 656 nm, as indicated by lines. Oxygen gives a high peak at 777 nm, suggesting that it exists as O I ion [20]. A comparison of Fig. 8, Fig. 9 clearly reveals that the height of these peaks measured during Ar injection is much larger than those for the air injection case. For example, when argon gas is used, the intensity of Hα peak reaches 80,000 count, while in the case of air, the peak intensity is only about 20,000 count. The third group of peaks corresponds to argon itself and, obviously, these peaks are observed only in the case of Ar injection in Fig. 9. These peaks have high intensity suggesting higher temperature in the argon-containing plasma that probably is a result of lower entropy of argon compared with air. This issue will be discussed in more details below. From this point of view, it becomes clear why the generation of chemically active radicals is enhanced when argon is introduced in the system. Thus, the above findings provide an additional explanation on the improved efficiency of RhB during the argon injection. It is to be noted that no OH radical related peaks were observed in the emission spectra. It is known that the OH related peak appears at a wavelength of 310 nm [21]. Hence, this peak is probably hidden by the broadened continuous spectra as shown in Fig. 8, Fig. 9.

### 4.3 Role of gas bubbles in the decomposition process

**Fig. 10:**
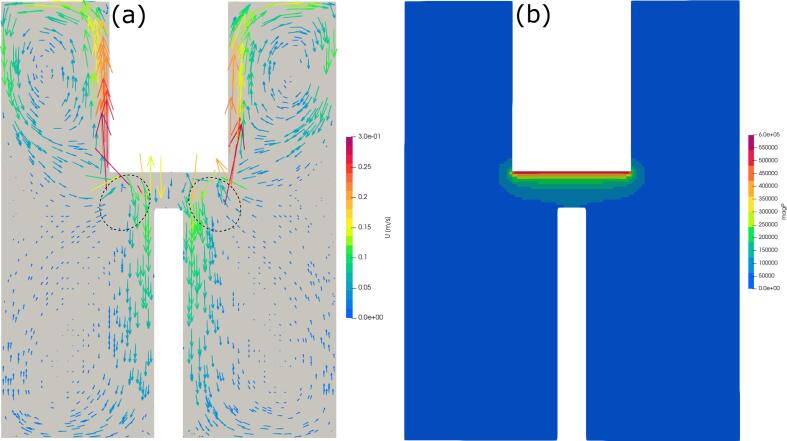
Velocity vector field (a) and distribution of sound pressure (b) in the vessel predicted numerically.

It can shown that small bubbles, formed during the injection of air or argon through the porous plug, are unable to enter into the cavitation zone in the interelectrode space because of high gradient of sound pressure there. Fig. 10 presents a vector field of acoustic streaming velocity (a) and sound pressure (b) in the reactor predicted numerically.

Simulation conditions were kept the same as those of experiments. Details on the numerical model have been reported in our previous paper [11]. It is readily seen that the liquid flow velocity in the vicinity of sonotrode tip is higher than the rising velocity of bubbles which was measured to be 0.2 m/s. Therefore, small bubbles are involved into the circulatory liquid flow moving down at the central part of the vessel and up near the vessel wall. Generally, the flow pattern allows some bubbles to be entrained into the cavitation zone, for example, at areas surrounded by the broken line. However, the strong primary Bjerknes force, acting on bubbles, pushes them out from the cavitation zone. As reported in [22], at sound wave antinodes the force is repulsive if |*P*| > 1.7 atm when the bubble radius is ranged from 3 to 5 μm. Also, bubbles with the diameter larger than equilibrium one (150 mm) are always subjected to the repulsive force because the oscillation phase between the ultrasound and bubble shifts by half cycle resulting in the repulsive force from antinode [23]. The predicted pressure distribution shows that the high-pressure amplitude zone with |*P*| > 1.7 atm is expanded widely in our experimental condition. Thus, this suggests that mm-sized bubbles cannot enter the cavitation zone and reach the interelectrode space during the ACAP and ultrasound alone treatments. On the other hand, the entrainment of small bubbles into the circulatory flow extends their residence in the liquid that is favorable for dissolution of gas in the liquid. Thus, one can conclude that, in the ACAP treatment process, the improvement in the RhB degradation efficiency with gas injection is associated with the gas dissolution. The phenomena of bubble entrainment and gas dissolution occur equally in air and argon injection. However, what makes a difference between air and argon is thermodynamic properties. Particularly, it is well known that the heat capacity ratio of argon is significantly greater than that of air [24]. Therefore, temperature inside argon bubble during its compression in ultrasound field should rise up to greater values compared to the air bubble case. As a result, formation of chemically active radicals in cavitating argon bubbles should occur more easily. This effect has been well known from the sonochemical literature, for example [25]. Higher temperature should raise the decomposition efficiency due to enhanced generation of chemically active radicals. The efficiency of argon injection can be clearly observed from Fig. 7(a), especially at larger flow rates, showing the emission spectra obtained during ACAP treatment combined with injection of air (Fig. 9) and Ar (Fig. 10). Injection was performed in a water bath through the porous plug at a flow rate of 8 L/min. Water conductivity and output voltage were kept constant at 100 μS/cm and 26 kV, respectively.

### 4.4 Comparison of various treatment methods

In this subsection, the ACAP process is compared with those when the plasma alone or ultrasound alone is applied with emphasis on the effect of gas flow rate and H_2_O_2_ formation. Comparison will be performed only for the argon injection through the porous plug because this combination was found to be the best among those examined. The assistance effect of ultrasound on the RhB degradation efficiency with electrical discharge plasma is more pronounced at a zero or low flow rate of argon injection. This can be readily seen from Fig. 7(a) and (b). Then, as the gas flow rate increases, the contribution of ultrasound to the RhB degradation becomes less while the influence of plasma remains significant. This is because argon, as it dissolves in water, begins to play an increasingly decisive role in the plasma generation through the above-mentioned mechanisms. On the other hand, in the case of ultrasound alone treatment, the presence of argon in water, although it enhances the degradation efficiency at lower gas flow rates, can have a negative effect at higher gas flow rates. This can be explained considering physical and chemical effects of ultrasound. In the present experiments, one of the main physical effects of ultrasound is acoustic streaming. As mentioned above, acoustic streaming is of prime importance in determining the flow pattern and mass transfer of RhB to the cavitation-assisted plasma zone. However, injection of gas at a high flow rate can cause the flow pattern to change in such a way that the mass transfer becomes slower. The main chemical effect of argon, as mentioned above, is an enhanced generation of active radical due to the greater heat capacity ratio of argon. Therefore, on the whole the dissolution of argon in water is favorable for sonochemical reactions. However, measurement of H_2_O_2_ formation rate reveals that the effect of argon on the radical production depends on the treatment method and conditions. Since a part of radicals, for example OH⋅, combine to produce H_2_O_2_, its formation rate may be a measure of radical formation activity. It is notable that H_2_O_2_ can be produced only from radicals under our experimental conditions. Our measurements showed that the rate of H_2_O_2_ formation in the RhB solution is 0.019 mg/min/L, 0.083 mg/min/L and 0.111 mg/min/L for the cases of ultrasound alone, plasma alone and ACAP treatment, respectively at the largest argon flow rate (8 L/min) examined. In experiments with pure water, the rate of H_2_O_2_ formation is 0.031 mg/min/L, 0.088 mg/min/L and 0.127 mg/min/L under the plasma alone, ultrasound alone and ACAP treatment, respectively. It is readily seen that the H_2_O_2_ formation rate during the ACAP treatment is slightly larger than the sum of H_2_O_2_ formation rates when only ultrasound or only plasma were used, and this tendency remains the same in both the RhB solution and pure water. These data clearly show that the effect of argon injection on the radical production during the plasma generation is more significant than that during the acoustic cavitation. Notice that the difference between the H_2_O_2_ formation rates measured in the RhB solution and water may provide an estimate for the amount of OH⋅ radicals consumed during degradation of RhB. The above results reveal that this amount is largest under the ACAP treatment. However, taking into account that the radical-involved chemistry is rather complicated and that concentration of H_2_O_2_ is utterly low, the accuracy of such estimates are generally rather low in quantitative terms.

Finally, it would be interesting to estimate the energetic efficiency of each treatment process. As mentioned above, the acoustic power was measured calorimetrically to be 116 W. Since all experiments were performed under the same conditions of ultrasound irradiation this value can be used for all estimates. As for the power of plasma discharge, this can be estimated as follows. As the plasma pulse is provided through the discharge of capacitor, the plasma power can be expressed in terms of electrical energy stored in the capacitor, E_C_ multiplied by the pulse frequency, f. The energy can be determined from Eq. (4)

(4) $$E_{C}=\frac{1}{2}CV^{2}$$

where C is the capacitor capacity, 1000pF and V is the voltage, 26 kV. The frequency was measured experimentally as explained above. The energetic efficiency can be defined in terms of amount of decomposed RhB per unit energy, and the unit energy amount can be expressed in kW⋅h, as suggested in [5]. The estimates are presented in Table 3 for the cases without argon injection and with it as the flow rate of 8 L/min.

**Table 3:** Energetical efficiency, g /kW⋅h.

Condition	ACAP	Plasma alone	Ultrasound alone
No Ar injection	0.11	N.a.*	0.08
Ar injection, 8 L/min	0.26	1.98	0.13

N.a : Not available because plasma pulse was not generated under this condition

These data reveal that the energetic efficiency of plasma alone treatment at high flow rate of argon is the best among the treatment methods considered. This is because the electrical energy of pulse discharge is converted in the thermal and chemical energy in a more efficient way compared to the acoustic energy case. However, when electrical pulse plasma is applied alone, significant consumption of argon is required that can lead to a rise in the cost of such a treatment.

## 5 Conclusions

In this study, we investigated the effect of gas injection on the efficiency of wastewater treatment in the ACAP process proposed by the authors earlier. The process combines the pulsed high-voltage discharge plasma with ultrasound vibrations introduced in a liquid bath through a sonotrode at a frequency of 20 kHz and acoustic power of 116 W. Experiments were performed using a batch reactor and a Rhodamine B (RhB) aqueous solution as a model contaminant. Air and argon were injected in the solution bath through L-shaped nozzles or a porous plug. Comparative experiments using plasma alone and ultrasound alone were also conducted. The results can be summarized in the following way.

(1) In general, injection of gas through the porous plug is more efficient than injection through L-shaped nozzles because the porous plug produces much finer bubbles that provides a wider surface area and longer residence time of bubbles in the solution.
(2) Argon provides a greater improvement in the RhB degradation efficiency compared to the air injection under all experimental conditions examined. The argon improvement effect is especially pronounced in the case of plasma treatment without ultrasound irradiation and becomes greater with increasing the argon flow rate.
(3) Under conditions using ultrasonic treatment alone, the gas injection has a significant effect on the RhB degradation efficiency at lower gas flow rates, but this effect is kept constant or slightly decreases as the gas flow rate increases.
(4) At the maximum flow rate of argon (8 L/min), the efficiency of RhB degradation is increased from 30% to 65% in the ACAP treatment, from 2% to 50% in the plasma-alone treatment, and from 20% to 30% in the ultrasound alone treatment.
(5) Plasma emission spectra revealed that chemically active hydrogen and oxygen-containing ions and radicals are generated in larger amounts when argon is introduced in the reactor compared to those during air injection.
(6) The degradation improvement effect of argon can be explained by the following three reasons. First, compared to the condition without gas injection, argon provides lowering the breakdown voltage to a greater extent than air does. As a result, at the working voltage examined, the frequency of plasma pulse discharge becomes higher when argon is injected compared to the air injection case. Second, argon has a higher solubility in water and lower entropy as compared to those of air. Third, argon does not lead to formation of by-product species which can inhibit the plasma discharge and RhB degradation.

## CRediT authorship contribution statement

**Yifan Xu:** Conceptualization, Methodology, Investigation, Validation, Visualization, Writing – original draft, Funding acquisition. **Takuya Yamamoto:** Formal analysis, Resources, Funding acquisition. **Daiki Hariu:** Methodology, Investigation, Validation, Data curation. **Sergey Komarov:** Supervision, Conceptualization, Funding acquisition, Project administration, Writing – review & editing.

## Declaration of Competing Interest

The authors declare that they have no known competing financial interests or personal relationships that could have appeared to influence the work reported in this paper.
